# Development of a novel technology for long-term culture and live imaging of excised human tissue

**DOI:** 10.1038/s41598-025-94022-0

**Published:** 2025-03-18

**Authors:** Takeshi Tohgasaki, Takayuki Sugimoto, Yoshika Sugimoto, Akira Takeda, Kyoko Baba

**Affiliations:** 1 FANCL Research Institute, FANCL Corporation, 12-13 Kamishinano, Totsuka-ku, Yokohama, Kanagawa Japan; 2 Department of Plastic and Aesthetic Surgery, Kitasato University School of Medicine, Sagamihara, Japan

**Keywords:** Live imaging, 4D imaging, Skin, Tissue culture, Biological techniques, Optics and photonics

## Abstract

An integrated understanding of the structure and reactivity of cells, extracellular matrices, and appendages is important for elucidating their functions and mechanisms in our bodies. Three-dimensional imaging using immuno-fluorescent staining with decolorization technology aids in comprehending the internal structure of human organs. However, live imaging of skin dynamics using animal models is highly invasive and unsuitable for humans. The aim of this study was to establish a non-invasive live imaging method for excised human tissue. In this study, to maintain excised human skin tissue in a state similar to a living body, we developed a novel microneedle-based culture technique. This method was evaluated for cytotoxicity detection, inflammatory cytokine release, and tissue morphology. Using microneedles, we cultured excised skin tissue and observed cellular organelles, reactive oxygen species (ROS), and fibrous structures via fluorescent probes and autofluorescence. The microneedle technique prevented cell death and inflammation, enabling long-term culturing. We live-imaged various skin cells, extracellular matrices, and appendage structures, visualizing epidermal cell membranes, mitochondria, and ROS. Collagen and elastin fibers were observed using autofluorescence and second harmonic generation. This approach enabled live imaging for 5 d, providing insights into skin metabolism, regeneration, and responses to stimuli and drugs, ultimately advancing dermatological research.

**Supplementary Information:**

The online version contains supplementary material available at 10.1038/s41598-025-94022-0.

## Introduction

Direct observation is crucial for understanding various phenomena within our bodies, such as aging, cancer, chronic inflammation, and wound healing. Decolorization technology in living organisms has facilitated the three-dimensional observation of whole bodies and organs at cellular-level resolution using optical microscopy, remarkably advancing our understanding of internal structures over the past decade^1–8^. However, as this technology requires sample fixation, it cannot be applied to live imaging, limiting our ability to investigate the process of decline in living organisms. Live tissue imaging techniques are required to enhance our understanding of tissue reactivity and alterations within tissues. In vivo imaging using multiphoton microscopy is effective for analyzing in vivo cellular dynamics across various tissues and organs^9–11^. In mice and rats, this technology contributes to understanding the interactions between osteoclasts and osteoblasts^12–17^, as well as the mechanisms of skin immunity^18–24^. Unfortunately, these methods are not applicable to humans because they require surgical exposure of organs under anesthesia and often rely on genetic engineering to generate fluorescence. Furthermore, clinical diagnostic tools such as computed tomography^25^, positron emission tomography^26^, magnetic resonance imaging^27^, and photoacoustic imaging^28–31^ provide deep, three-dimensional images of the living body. However, these techniques lack the resolution needed for single-cell level analysis and encounter challenges in long-term live imaging because of imaging time, cost, and invasiveness. Observing human organs and tissues at the single-cell level non-invasively and over extended periods remains challenging. Therefore, we aimed to develop a method that enables live imaging of cultured human tissue with cellular-level resolution over a long period as an alternative approach for examining the internal structure of human organs.

We focused on human skin tissue, selecting it as a target for live imaging of excised organs. Human skin tissue is relatively easy to obtain, often available in surplus through cosmetic surgery. Human skin features a layered structure comprising the epidermis, dermis, and subcutaneous tissue, each with distinctive cell types, extracellular matrices, and appendages, exhibiting unique structures and dynamic behaviors. The epidermis is abundant in keratinocytes and undergoes active turnover through keratinization. The dermis contains an abundance of extracellular matrix and various appendages such as hair follicles, blood vessels, and nerves. The subcutaneous tissue contains adipocytes, immune cells, and blood vessels. Based on these facts, we believed it would be possible to observe various living phenomena by observing the excised skin.

However, the challenge is to maintain the excised tissue in a state similar to that of a living tissue for a long period. Typically, skin tissue is cultured using a cell insert to draw the culture medium up from the lower dermis. However, this conventional method often causes cell necrosis and separation of the epidermis and dermis within a few days of removal. In large-volume spheroids and organoids, central necrosis occurs as a result of limitations in material exchange, such as insufficient oxygen and nutrient supply or waste accumulation^32–34^. Cell death and tissue breakdown in excised skin are believed to result from the same issues that lead to internal necrosis in spheroids and organoids.

Therefore, in this study, we devised a new culture method for long-term culture using microneedles. This approach would ensure an adequate nutrient supply, waste removal, and gas exchange in thick, living skin tissues, facilitating long-term time-lapse imaging. In this paper, we describe a novel tissue culture method that enables long-term live imaging of cells, extracellular matrices, blood vessels, hair follicles, and adipocytes within skin tissue.

## Results

### Microneedles are useful for low-invasive, long-term culture of skin tissue

**Fig. 1:**
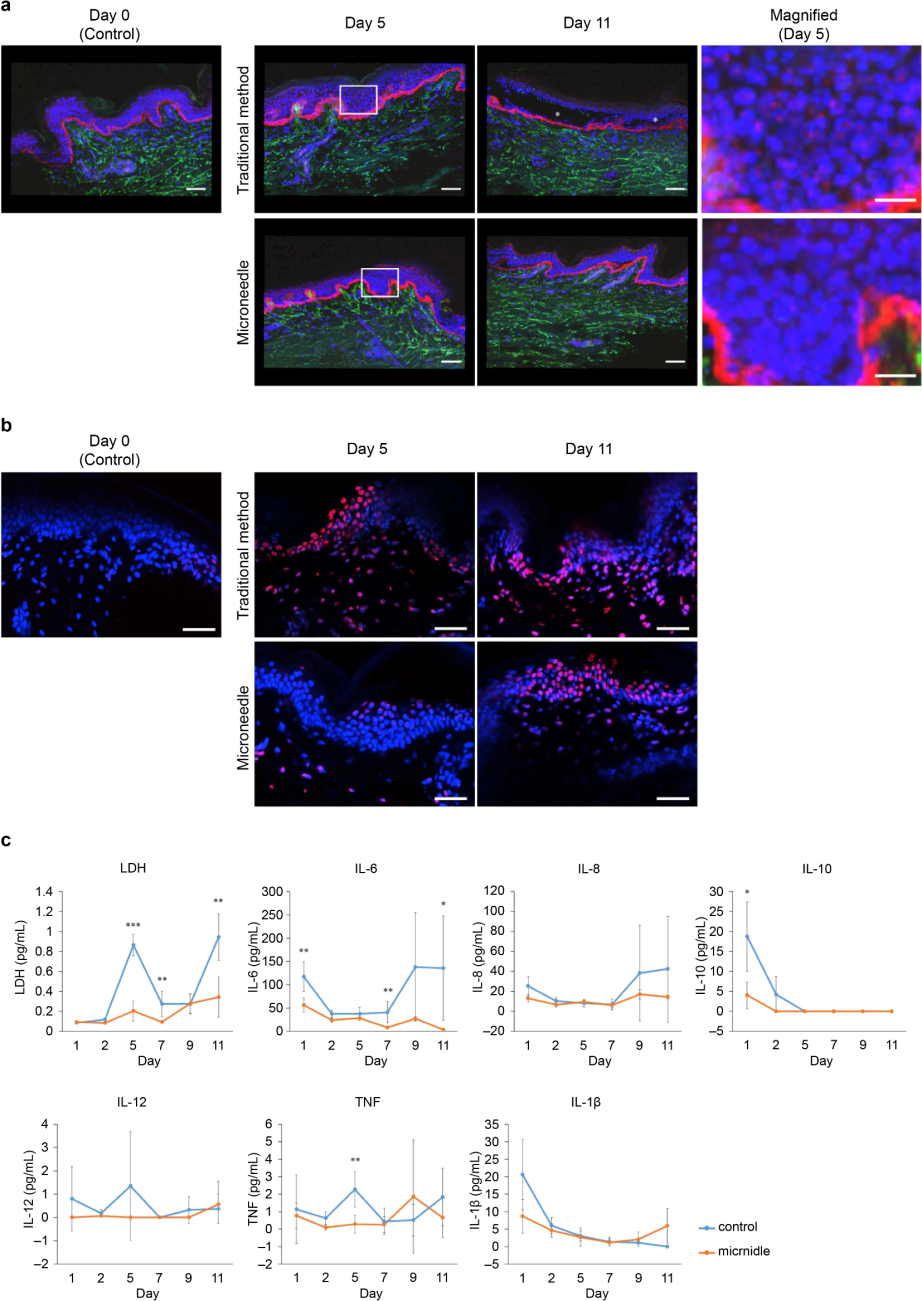
Comparison of changes over time in skin tissue with each culture method. (**a**) Fluorescent immunostaining images of skin tissue after 0, 5, and 11 d of culture. Day 0 represents the state immediately after extraction and before culturing, serving as the control. The traditional method (upper row) and microneedle method (lower row) are displayed. In skin tissue cultured using traditional methods, the epidermis and dermis become exfoliated. The white asterisk on Day 11 indicates the region where the epidermis and dermis of the traditionally cultured skin tissue have peeled away. Each white bar indicates a scale of 100 μm. The image on the right provides a magnified view of the white frame in the Day 5 images. The nuclei of epidermal cells in traditionally cultured skin tissue appear smaller and nearly perfectly circular. Blue: nucleus; green: tropoelastin; red: COL VII. Each white bar indicates a scale of 50 μm. (**b**) TUNEL staining of skin tissue after 0, 5, and 11 d of culture. Day 0 represents the state immediately after extraction and before culturing, serving as the control. The traditional method (upper row) and microneedle method (lower row) are displayed. In skin tissue cultured using traditional methods, the epidermis and dermis become exfoliated. Blue: nucleus; red: TUNEL. Each white bar indicates a scale of 50 μm. (**c**) Concentrations of LDH and inflammatory cytokines in the culture medium supernatant following skin tissue culture. Gray line: traditional method; black line: microneedle culture. Bars represent S.D. (*n* = 3). *p < 0.05, **p < 0.01, and ***p < 0.001.

We compared the conventional method of medium infiltration through a polycarbonate membrane into the lower dermis with a technique that uses microneedles to inject the medium directly into the skin. After 5 d of culture with the traditional method, the nuclei of epidermal cells appeared smaller and nearly perfectly circular, indicating cellular damage. However, this was not observed with the microneedle method. Additionally, by the 11th day of traditional culture, the epidermis and dermis had separated, whereas the layers remained bonded using the microneedle method (Fig. 1a). Furthermore, the degree of DNA damage was detected by TUNEL staining. Almost no DNA damage was observed in the skin tissue before and after 5 d of culture with microneedles. However, DNA damage was observed in both epidermal and dermal cells in the skin tissue after 5 and 11 d of culture using the conventional method. DNA damage was also confirmed in the epidermis of skin tissue after 11 d culture by microneedle. Still, it was less in the dermis (Fig. 1b). Moreover, levels of LDH, IL-6, IL-10, IL-1beta, and TNF in the supernatant of skin tissues cultured using microneedles were significantly lower than in those cultured using the conventional method (LDH: *p* = 0.023 [Day 2], *p* = 0.002 [Day 5], *p* = 0.015 [Day 7], and *p* = 0.015 [Day 11]; IL-6: *p* = 0.017 [Day 1], *p* = 0.001 [Day 5], *p* = 0.002 [Day 7], and *p* = 0.0003 [Day 9]; IL-10: *p* = 0.011 [Day 1]; and TNF: *p* = 0.012 [Day 5] and *p* = 0.036 [Day 9]) (Fig. 1c). Although IL-12 levels and some other time points were not significantly different, they tended to be lower when using microneedles for culture. However, the level of IL-1β did not differ significantly on day 11 (*p* = 0.053), but it tended to be higher when microneedles were used for culture.

### Time-lapse imaging of skin appendages and fatty tissue

The excised human tissue of skin with subcutaneous fat was observed using the novel culture method described above. The cell nuclei, cell membranes, mitochondria, microtubules, and ROS of keratinocytes and each dermal or fatty cell in human skin tissue were labeled with fluorescent probes. Each fluorescent probe was effective in the skin tissue, and each target was visualized. Additionally, the autofluorescence of collagen and elastin fibers was detected across a broad range of approximately 425–550 nm. We conducted time-lapse imaging of appendages such as capillaries and hair follicles in human skin tissue, as well as adipocytes in subcutaneous fat tissue. We successfully captured time-lapse images of capillaries and surrounding smooth muscle cells (Video 1) and immune cell-like cells (Video 2), cells forming hair follicles and surrounding fibrous elastin and collagen (Video 3), and the fatty cells and the surrounding capillaries (Video 4) in the dermis and subcutaneous tissue.

### Time-lapse imaging of the epidermal–dermal junction in excised human skin tissue

**Fig. 2:**
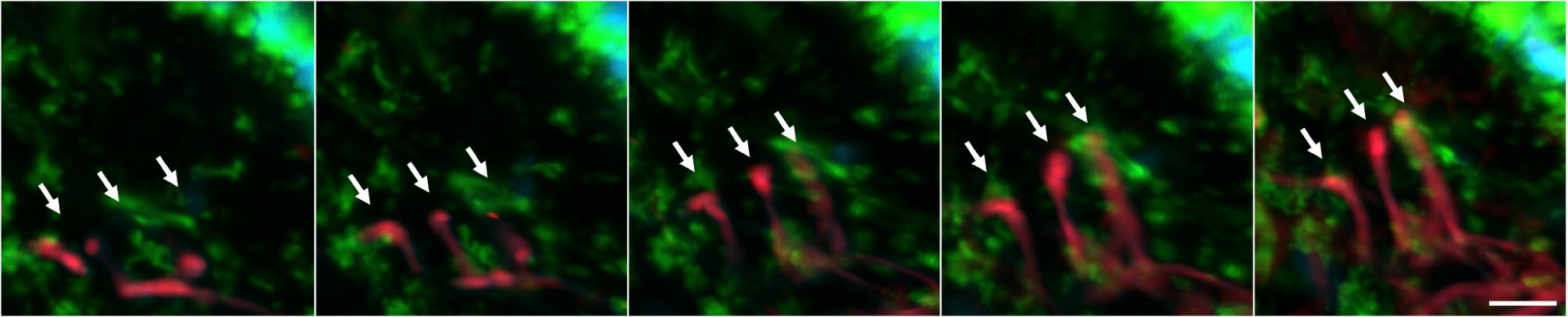
Elongation of dermal elastic fibers. Three oxytalan fibers extend vertically from elaunin fibers in the dermis toward the epidermal basement membrane (white arrows). Images excerpt from Video 7. Blue: cell nucleus, green: cell membrane, red: elastin fibers (autofluorescence). A white bar indicates a scale of 10 μm.

The epidermal–dermal junction in the excised human skin tissue was observed using the novel culture method described above. In the dermis, cells forming clusters and cells migrating within the matrix were observed, with cells migrating around and between clusters being particularly noticeable. However, no dynamic movement, such as cell migration or morphological changes, was observed in the epidermis (Video 5). Regarding the levels of ROS in the epidermis and dermis, epidermal cells showed unique ROS levels in each cell. In the dermis, autofluorescence of the extracellular matrix was observed, but intracellular ROS was hardly detected (Video 6). Additionally, extending fibers were observed perpendicularly to the basement membrane from elastin fibers running parallel to the basement membrane in the dermis (Fig. 2, Video 7). Furthermore, at the epidermis–dermis boundary, it was observed that the dermal-side cell membranes of the epidermal cells on the basement membrane extend toward the dermis, and the cells of the dermis extend toward the basement membrane, respectively, and communicate to each other (Video 8).

### Time-lapse observation of membrane movement of basal layer cells

**Fig. 3:**
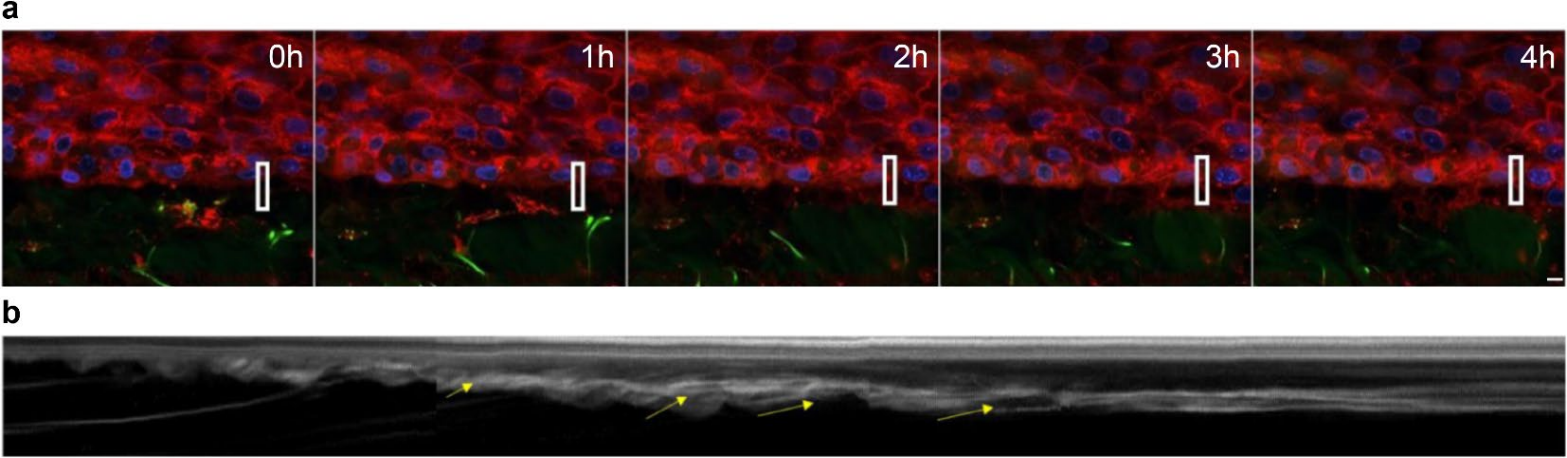
Velocity analysis of the retrograde flow of epidermal cells at the epidermis–dermis junction. (**a**) Overall image of the epidermis–dermis boundary at 0, 1, 2, 3, and 4 h (Video 2). Blue indicates the nucleus, green shows the autofluorescence of elastin fibers (high intensity) and collagen fibers (low intensity), and red represents the cell membrane. A white bar indicates a scale of 5 μm. (**b**) A kymograph, created by isolating the white-framed area in the overall image, converts the cell membrane (red) to grayscale and arranges it chronologically. The retrograde flow velocity is calculated based on the angle of the diagonal line.

In addition, time-lapse observations at a high resolution of approximately 0.2 μm/pixel and 0.5 frames/s were performed at the epidermal–dermal junction for 4 h using a 100× oil immersion lens and 2× digital zoom. It was observed that the epidermal cell membranes on the basal layer extended toward the dermis and pulled in fibers and cells in the dermis (Video 9). At this time, retrograde flow-like membrane movement toward the cell center was observed in the cell membranes of epidermal cells on the basement membrane. We measured the speed of this retrograde flow-like flow by analysis using a kymograph, revealing a flow rate ranging from 2.4 to 5.8 μm/min (Fig. 3).

## Discussion

In this study, we explored a long-term live imaging method of excised human skin at the cellular level. First, we developed a new culture technique to facilitate prolonged imaging while preserving the original biological functions of the excised skin tissue. Spheroids and organoids are known to be unsuitable for long-term culture because of necrosis arising from their limited capacity for material exchange^32–34^. Conventional culture methods that rely on infiltrating the medium into excised skin tissue were ineffective at preventing cell death within the skin and disrupted the epidermis–dermis junction. To address this issue, we devised a technique that uses microneedles to infuse the culture fluid directly into the excised skin tissue. Although a non-significant increase in IL-1β was detected on day 11 from starting the culture of the microneedle, compared to conventional culture methods, our approach significantly reduced the levels of LDH, IL-6, IL-10, and TNF. Additionally, the morphology of the cell nucleus indicated a reduction in cytotoxicity, and TUNEL staining suggested that damage inside the skin tissue would be decreased by microneedle culture. Furthermore, the conventional method led to a separation of the epidermis–dermis junction, whereas our method maintained the connection at 11 d after the start of culture. These results suggest that microneedles promote substance circulation and prevent necrosis and toxicity by infusing the culture medium directly into the skin, thereby preserving the function of the excised skin tissue over an extended period.

We utilized this culture method to conduct long-term time-lapse imaging of the behavior, condition, and morphology of cells in the epidermis, dermis, and appendages, such as blood vessels and hair follicles in excised skin tissues. The efficacy of fluorescent probes in the visualization of cell nuclei, cell membrane ROS, mitochondria, and microtubules was verified even within excised skin tissue. Furthermore, fibrous elastin and collagen emitted autofluorescence with a long fluorescence spectrum. Despite the overlapping wavelengths of autofluorescence and the fluorescence of each organelle labeled with the probe, the structure could still be distinguished from its shape in the majority of cases. As a result, we succeeded in time-lapse observation of appendage structures such as blood vessels, hair follicles, and subcutaneous fat inside the excised skin at the single-cell level. We captured cell migration within the dermis. Cells in the epidermis and dermis were labeled with a fluorescent indicator to detect ROS levels. Observations revealed individual variations in ROS levels, with higher concentrations in cells on the basement membrane and lower concentrations in dermal cells. These findings confirm that cells remain viable in excised skin tissue, enabling time-lapse imaging of living tissue. We also noted that structures extending from the basement membrane into the dermis moved actively, whereas dermal structures were drawn toward the epidermis–dermis interface. We also succeeded in capturing retrograde flow-like dynamics of the cell membrane and measuring the speed of this flow. This live imaging approach for excised tissue, with hundreds of nanometers of spatial resolution and second-level temporal resolution, aids the analysis of cellular behavior, structure, and molecular dynamics. Our imaging technique enables analysis at the single-cell level, which was previously not attainable using conventional methods in human studies and enables us to verify in vitro findings from cultured cells. Moreover, unlike most conventional in vivo observations, which are limited to a single time point, this method enables time-lapse observations, significantly enhancing our understanding of various phenomena over time.

In addition to the reported potential chemical interactions between the epidermis and dermis^35^, these imaging data suggest an active physical interaction between the two layers. Analysis of this region using live imaging could advance the development of mechanisms and treatments related to the binding between COLVII and elastin fibers, epidermolysis bullosa, and wound healing. Moreover, this approach captured the structures of capillaries, hair follicles, adipocytes, and various cells surrounding these appendages, such as smooth muscle cells. It also recorded the movements of basement membrane protrusions and immune cells in their living state. Preliminary tests using excised skin tissue from other donors showed a decrease in cytotoxicity and inflammatory cytokine concentrations (data not shown) using the microneedle culture method. However, human tissue varies between individuals and depends on surgical conditions, raising uncertainty regarding the likelihood of obtaining consistent results from all skin tissues. Additionally, this study only evaluated tissues up to 11 d after culture, with longer-term effects remaining unexamined. Although this study assessed cytotoxicity, inflammatory cytokines, and tissue morphology, the impact of different culture methods on gene and protein expression and skin functions remains unknown. The reduced cytotoxicity observed with the microneedle culture method could be attributed to the supply of nutrients or the removal of waste products from the internal tissue, but the exact mechanism remains unclear. Further investigation into the underlying mechanism is required to improve culture methods. Additionally, the phenomena observed in excised tissue might differ from those in the living body, necessitating further verification to determine if the findings in excised tissue also occur in vivo.

In this study, we demonstrated that microneedles suppress inflammation and cytotoxicity in excised human skin tissues, contributing to long-term culture and live imaging. This microneedle culture method could also be applied to other excised organs. Moreover, through long-term live imaging of various structures within excised human skin tissue, we captured phenomena observable only in living tissue, such as cellular and tissue states and movements. Using this imaging method, we anticipate gathering insights into metabolic wound healing mechanisms within the skin and evaluating the responsiveness to drugs. Furthermore, microneedles are expected to be applied to medical treatments as painless and minimally invasive drug administration into the skin. The relationship between the material and shape of microneedles, the depth and layer of drug delivery, and the drug release rate have been studied^36^. The method in this study can also be applied to microneedle efficacy evaluation. Our technique will contribute to advancing dermatological research and developing novel treatments for skin conditions.

## Materials and methods

### Human skin tissue samples

We used unfixed human excised abdominal skin tissue obtained from BIOPREDIC International (Rennes, France) to verify the impact of the culture method with and without microneedles (as described later). For live imaging purposes, excised abdominal skin tissue was provided by the University School of Medicine. The study protocol adhered to the ethical guidelines of the University’s Ethics Committee (approval number: B20-333) and the Ethics Committee of the FANCL Co., Ltd. (approval number: C2020-039). The research was conducted in accordance with the principles of the Declaration of Helsinki. Written informed consent was obtained from each patient before study enrollment.

### Culture of human skin tissue for evaluation of culture methods

Each skin tissue excised from the human abdomen was transported at 4 °C to the laboratory within 24 h. After arrival, unfixed human skin tissues were immediately cut into 9 mm-diameter and 900 μm-thick pieces and used to start a culture employing two different methods. For the conventional osmosis culture (control), each skin piece was placed in a cell culture insert (Thermo Fisher Scientific, Waltham, MA, USA), allowing the medium to permeate from the bottom through a polycarbonate membrane. In contrast, for the infusion culture, in addition to the above methods, a microneedle was inserted into a piece of skin through the epidermal surface, and a medium was injected into the skin. In the former method (control), 4 mL of Dulbecco’s Modified Eagle Medium: Nutrient Mixture F-12 (DMEM F-12) (Thermo Fisher Scientific) was placed in the 6-well plate (Sumiron Co., Ltd., Osaka, JAPAN), which is a cell insert tray. For the latter, 2 mL of DMEM F-12 was placed in the 6-well plate, and 2 mL of DMEM F-12 was injected into the skin tissue via the microneedle. The cultures were maintained at 37 °C and 5% CO_2_. The culture medium was collected and replaced on days 1, 2, 5, 7, 9, and 11. Each skin sample was fixed in 4% paraformaldehyde in PBS before or after 5 and 11 d of culture. This culture method was based on the protocol for the NativeSkin^®^ (Genoskin, Toulouse, France) model. Still, the added volume of the medium was slightly greater than in the original protocol, considering the frequency of the replacement of the medium.

### Microneedle

**Fig. 4:**
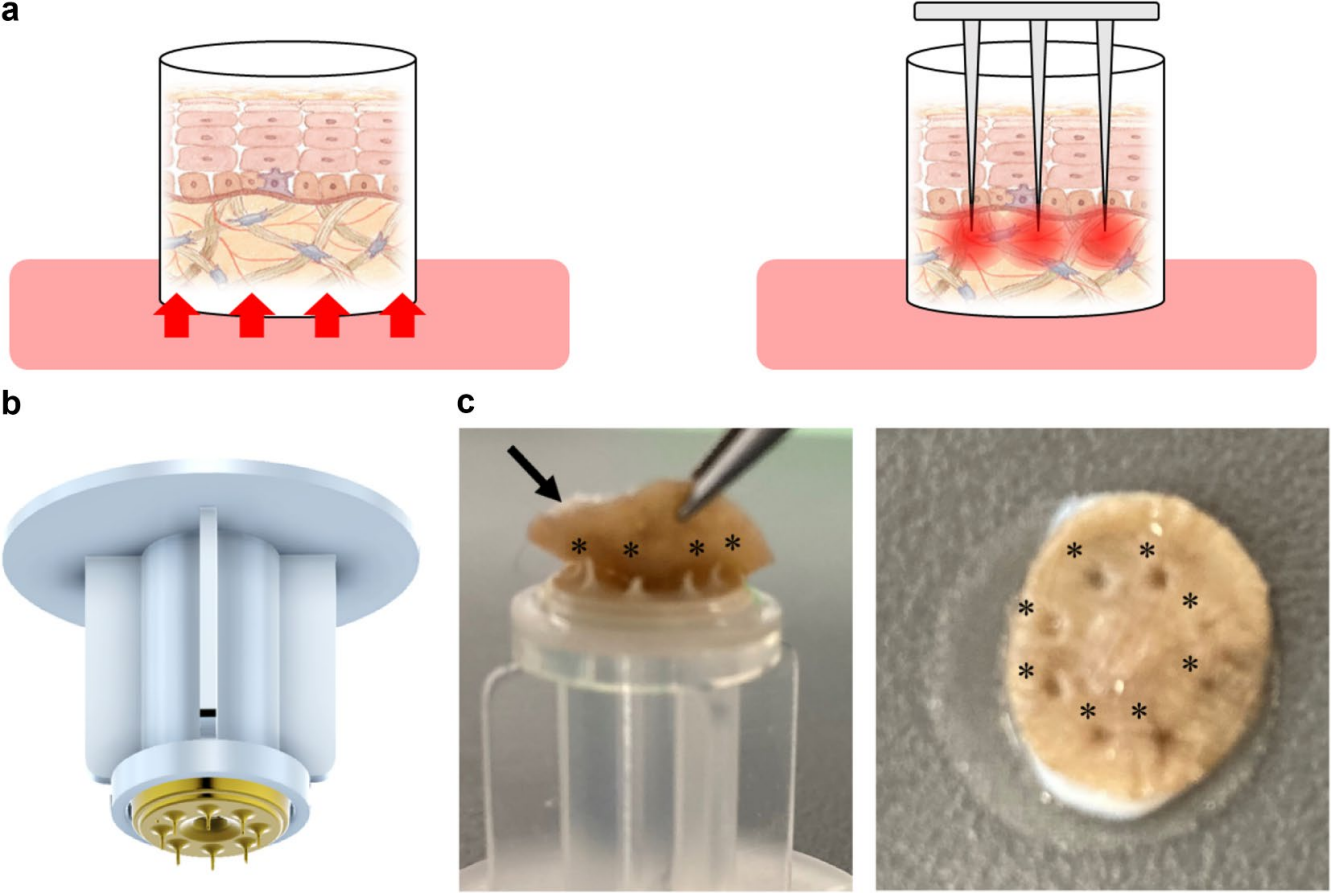
Schematic diagrams of each culture method and actual images of microneedles. (**a**) In the traditional culture method, skin tissue is placed in a cell insert, and the culture medium infiltrates from the dermal side through a polycarbonate membrane (left). In the culture method using microneedles, several needles with a diameter of 0.1 mm and a length of 1.0–1.2 mm are inserted into the dermis of the skin tissue (right). (**b**) A photograph of microneedles. An attachment fitted with eight microneedles. (**c**) Photograph fitting to the excised skin tissue (left). Eight microneedles (black asterisk) were inserted into the excised skin tissue (black arrow). Photo after insertion (right). Photograph taken from the surface of the skin (right). A black asterisk indicates the location of the inserted trace.

The microneedles used for the culture were provided by Think-Lands Co., Ltd. (Kawasaki, Japan, https://www.think-lands.co.jp/). To ensure compatibility with the strength and thickness of the excised skin tissue and to prevent needle clogging, we developed a disk-shaped component featuring eight microneedles with diameters of 0.1 mm and lengths of 1.0–1.2 mm (Fig. 4). This needle component was attached to the tip of an infusion tube and inserted from the epidermal side of the skin, allowing fluid infusion into the skin tissue via the eight needles. The infusion rate was regulated using a syringe pump (AS ONE CORPORATION, Osaka, Japan).

### Immunofluorescence staining and TUNEL staining

Each fixed skin tissue sample was sliced into 1 mm-thick sections oriented along the epidermis–dermis axis. The sections were washed with 0.05% Tween 20 in PBS and blocked with Starting Block blocking buffer (Thermo Fisher Scientific). They were then labeled with anti-tropoelastin mouse monoclonal antibody (Santa Cruz Biotechnology, Dallas, TX, USA) and anti-COL VII rabbit polyclonal antibody (Abcam, Cambridge, MA, USA), both diluted to 1:500 in the Starting Block blocking buffer. After washing, the sections were stained with Goat Anti-Mouse IgG H&L Alexa Fluor^®^ 488 and Goat Anti-Rabbit IgG H&L Alexa Fluor^®^ 647 (Thermo Fisher Scientific) at a 1:1000 dilution. Nuclear staining was performed using DAPI (DOJINDO Lab, Kumamoto, Japan) at a 1:5000 dilution. Detection of DNA damage was performed using the TUNEL Assay Apoptosis Detection Kit (Biotium, San Francisco, CA, US). Finally, after washing, the sections were decolorized using Rapiclear 1.49 (SunJin Lab Co., Hsinchu, Taiwan).

### Measurement of cytotoxicity and inflammatory cytokines

To assess the effectiveness of the culture method using microneedles, lactate dehydrogenase (LDH) and inflammatory cytokines (IL-6, IL-8, IL-10, IL-12, IL-1β, and TNF) in the collected culture medium were measured using an LDH measurement kit (Fujifilm Wako Pure Chemical Industries, Ltd.) and a Cytometric Bead Array (CBA) Human Inflammatory Cytokine Kit (Becton, Dickinson and Company).

### Imaging

Nuclei, tropoelastin, and COL VII in fixed and decolorized skin tissue were observed in three dimensions using a confocal laser scanning microscope (A1R, Nikon Solutions, Japan). In unfixed excised human skin tissue, nuclei, cell membranes, ROS, mitochondria, and microtubules were fluorescently labeled with NucBlue Live Cell Stain (Invitrogen, MA, USA), CellMask (Thermo Fisher Scientific), CM-H2-DCFDA (Thermo Fisher Scientific), MitoTracker (Thermo Fisher Scientific), and SPY650-Tubulin (Cytoskeleton, Inc., CO, USA), respectively, at a 1:1000 dilution. Each fluorescent label, along with the autofluorescence of collagen and elastin fibers, was observed using a confocal laser microscope (A1R or AXR, Nikon Solutions). A microscope stage culture device with an attachment for inserting microneedles into skin tissue (TOKAI HIT, Japan) was developed and employed for long-term live imaging.

### Statistical analysis

Differences between groups were assessed using Student’s *t*-test with BellCurve for Excel ver. 7.0 (Social Survey Research Information Co. Ltd., Tokyo, Japan). Results with *p* values < 0.05 were considered statistically significant.

## Electronic supplementary material

Below is the link to the electronic supplementary material.

Supplementary Material 1

Supplementary Material 2

Supplementary Material 3

Supplementary Material 4

Supplementary Material 5

Supplementary Material 6

Supplementary Material 7

Supplementary Material 8

Supplementary Material 9

Supplementary Material 10

## Data Availability

The data that support the findings of this study are available from the corresponding author, upon reasonable request.

## Abbreviations

CBA: Cytometric bead array
DMEM F-12: Dulbecco’s modified Eagle medium: nutrient mixture F-12
LDH: Lactate dehydrogenase
PBS: Phosphate-buffered saline
ROS: Reactive oxygen species
