# Supplementary material for: Development of a novel technology for long-term culture and live imaging of excised human tissue
Source: Sci Rep. 2025 Mar 18;15:9259. doi: 10.1038/s41598-025-94022-0 (PMC11920518; doi:10.1038/s41598-025-94022-0)
Supplement: Supplementary file 10 — Supplementary Material 10 [file 41598_2025_94022_MOESM10_ESM.docx]

**VIDEO LEGENDS**

**Video 1**. Time-lapse imaging of capillary vessels and surrounding smooth muscle cells in the dermis of excised skin tissue. Blue: nucleus; green: cell membrane and autofluorescence of elastin fibers (high intensity) and collagen fibers (low intensity); red: mitochondria. A white bar indicates a scale of 40 μm. The white text in the upper left corner indicates the time (hh:mm).

**Video 2**. Time-lapse imaging of capillary vessels and immune cells in the dermis of excised skin tissue. Blue: nucleus, autofluorescence of elastin fibers (high intensity) and collagen fibers (low intensity); green: cell membrane; re: mitochondria. A white bar indicates a scale of 10 μm. The white text in the upper left corner indicates the time (hh:mm).

**Video 3**. Time-lapse imaging of hair follicles in the dermis of excised skin tissue. Blue: nucleus, autofluorescence of elastin fibers (high intensity) and collagen fibers (low intensity); green: cell membrane; red: microtubules. A white bar indicates a scale of 50 μm. The white text in the upper left corner indicates the time (hh:mm).

**Video 4**. Time-lapse imaging of adipocytes and surrounding capillary vessels in the subcutaneous tissue of the excised skin tissue. Blue: nucleus; green: cell membrane and autofluorescence of elastin fibers (high intensity) and collagen fibers (low intensity); red: microtubules. A white bar is provided to show a scale of 50 μm. The white text in the upper left corner indicates the time (hh:mm).

**Video 5**. Migration of cells within the dermis of the excised skin tissue. Blue: cell nucleus, green: cell membrane, red: mitochondria and elastic fibers (autofluorescence). A white bar indicates a scale of 50 μm. The white text in the lower right corner indicates the time (hh:mm:ss).

**Video 6**. ROS imaging in epidermal and dermal cells of the excised skin tissue. The merged image (left). Blue: nucleus; green: ROS, autofluorescence of elastin fibers (high intensity) and collagen fibers (low intensity); red: cell membrane. Image processed to highlight ROS levels in epidermal cells (right). Thermoscale: ROS levels in epidermal cells. White: structures in the skin. A white bar indicates a scale of 30μm. The white text in the upper left corner indicates the time (hh:mm).

**Video 7.** Elongation of elastin fibers in the dermis of excised skin tissue. Blue: cell nucleus, green: cell membrane, red: mitochondria and elastic fibers (autofluorescence). A white bar indicates a scale of 5 μm. The white text in the lower right corner indicates the time (hh:mm:ss).

**Video 8**. Movement of the elongated structure of the basement membrane of excised skin tissue. Blue: nucleus; green: autofluorescence of elastin fibers (high intensity) and collagen fibers (low intensity); red: mitochondria. A white bar indicates a scale of 5 μm. The white text in the lower right corner indicates the time (hh:mm:ss).

**Video 9**. Retrograde flow of the epidermal cell membrane at the epidermis–dermis junction of excised skin tissue. Blue: nucleus; green: autofluorescence of elastin fibers (high intensity) and collagen fibers (low intensity); red: cell membrane. A white bar indicates a scale of 5 μm. The white text in the upper left corner indicates the time (hh:mm).
